# Clinical impact of tetracyclines and/or proton pump inhibitors on the efficacy of epidermal growth factor receptor inhibitors in non-small cell lung cancer: a retrospective cohort study

**DOI:** 10.1186/s12885-023-10623-w

**Published:** 2023-02-13

**Authors:** Hui-Hsia Hsieh, Tien-Yuan Wu, Chi-Hua Chen, Yu-Hung Kuo, Mann-Jen Hour

**Affiliations:** 1 Department of Pharmacy, Taichung Tzu Chi Hospital, Buddhist Tzu Chi Medical Foundation, Taichung, Taiwan; 2 School of Pharmacy, China Medical University, Taichung, Taiwan; 3 Graduate Institute of Clinical Pharmacy, Tzu Chi University, Hualien, Taiwan; 4 Department of Research, Taichung Tzu Chi Hospital, Buddhist Tzu Chi Medical Foundation, Taichung, Taiwan

**Keywords:** Epidermal growth factor receptor-tyrosine kinase inhibitors, Tetracyclines, Proton pump inhibitor, Non-small cell lung cancer

## Abstract

**Background:**

This retrospective cohort study examined the impact of tetracyclines (TCs) and proton pump inhibitors (PPIs) alone or in combination on the efficacy of epidermal growth factor receptor tyrosine kinase inhibitors (EGFR-TKIs) in patients with non-small cell lung cancer (NSCLC).

**Methods:**

Patients with NSCLC treated with gefitinib or erlotinib for at least 1 week between January 2009 and October 2021 were enrolled and divided into four groups based on the presence/absence of TC and/or PPI in the therapeutic regimen: TC-/PPI-, TC + /PPI-, TC-/PPI + , TC + /PPI + . Progression-free survival (PFS) and overall survival (OS) were the primary and secondary endpoints, respectively.

**Results:**

The estimated median PFS and OS of 347 included patients with NSCLC were 8.57 (95% confidence interval [CI]: 7.66–9.48) months and 13.10 (95% CI: 11.03–15.17) months, respectively. Co-administration of EGFR-TKIs with PPIs decreased the PFS and OS, while that with TCs improved the PFS and OS. However, the concomitant use of EGFR-TKIs, TCs, and PPIs yielded survival rates similar to that of EGFR-TKI therapy alone.

**Conclusions:**

The administration of EGFR-TKIs with other drugs poses a challenge in managing patients with NSCLC. Therefore, reassessing the indications and necessity of TC or PPI therapy is essential for patients receiving erlotinib or gefitinib. The benefits and risks of possible discontinuation due to the clinical relevance of this interaction should be considered.

## Background

Lung cancer is the leading cause of cancer-related death in men and women worldwide. This disease was expected to result in the deaths of nearly 132,000 individuals in the USA in 2021 [1]. Almost 85% of lung cancers are non-small cell lung cancer (NSCLC), which can be classified into adenocarcinomas, large cell carcinomas, and squamous cell carcinomas based on histopathology [2]. Nearly half of lung cancers are discovered at an advanced stage and have a poor prognosis [3]. The medical management of advanced NSCLC includes chemotherapy, targeted therapy, or immune checkpoint blockade therapy. Gefitinib, erlotinib, osimertinib, and afatinib are among the epidermal growth factor receptor (EGFR) tyrosine kinase inhibitors (TKIs) approved by the Food and Drug Administration for the treatment of metastatic NSCLC. The tumor tissue of all patients with NSCLC should be assessed for mutations as they frequently present with EGFR exon 19 deletions or exon 21 (L858R) mutations. EGFR mutations have been revealed to be predictors of the efficacy of EGFR-TKIs [4].

EGFR-TKIs have been tolerated well by most patients in clinical trials, and less than 10% discontinued treatment because of adverse events. EGFR is a transmembrane glycoprotein expressed in normally proliferating keratinocytes in the epidermis's basal layer and the hair follicle's outer layer. EGFR plays a critical role in the epidermis, stimulating epidermal growth, inhibiting differentiation and inflammation, and accelerating wound healing. Skin toxicity is the most common adverse effect associated with EGFR-TKI therapy (approximately 50–80%), which mainly includes acneiform eruption, papulopustular eruption, or pruritus. The eruption onset usually occurs within 2–4 weeks of TKI initiation but may also occur earlier or later [5]. Several studies have linked acneiform rash to increased survival or objective response rates, which may be a marker of the efficacy of the EGFR inhibitor [6]. A meta-analysis of 24 trials where patients took erlotinib or gefitinib found that mortality was lower in patients who developed skin rashes than in those without skin rashes, which also predicted the disease progression-free outcomes [7]. Acneiform rash caused by EGFR-TKIs can be treated with oral tetracyclines (TCs) for 4–6 weeks, but no clinical trial has demonstrated if antibiotics can improve the survival rate in EGFR-TKI users [8].

Up to 33% of patients with cancer use proton pump inhibitors (PPIs), the most commonly used gastric acid suppressants worldwide [9]. PPIs reduce gastric acid secretion by inhibiting H + /K + -ATPase and relieve stomach syndrome caused by anticancer drugs. Currently, there is a paucity of data on the safety of the concomitant use of PPIs and anticancer drugs [10]. In fact, gastric acid suppression induced by PPIs reduces the effectiveness of drugs that require an acidic environment for absorption.

Erlotinib solubility in the stomach is pH-dependent, and concomitant use with gastric acid suppressants may cause decreased solubility and absorption of erlotinib [11–13]. Therefore, concurrent use is not recommended in clinical practice. Several studies have shown that erlotinib is less effective when co-administered with gastric acid suppressants. The first study to demonstrate adverse effects conducted a retrospective analysis of 507 patients with advanced NSCLC, 124 of whom were also taking gastric acid suppressants. The study found that the median progression-free survival (PFS) was 1.4 months versus 2.3 months (hazard ratio [HR] 1.83, *p* < 0.001), and the overall survival (OS) was 12.9 months versus 16.8 months (HR 1.37, *p* = 0.003) in gastric-acid suppressant users versus non-users [14].

Gefitinib solubility decreases in a high pH environment. Therefore, the use of gastric acid suppressants will increase the pH of the stomach and consequently lead to decreased absorption and bioavailability of gefitinib [13]. Studies have shown that co-administration of omeprazole reduces the area under the curve and maximum serum concentration of gefitinib [15].

EGFR-TKIs are expensive targeted therapy drugs, and patients who develop rashes may have a better response and prognosis to EGFR-TKIs [6, 7]. None of the prospective trials conducted to date have included a sufficient study population to demonstrate the effect of TCs used for patients with skin rashes on the survival rate. Additionally, an increased gastric pH may reduce the absorption of TKIs, although limited evidence is available on this well-known clinical impact. This study examined the impact of TCs and PPIs alone or in combination on the efficacy of EGFR-TKIs in patients with NSCLC.

## Materials and methods

### Ethics approval

The study has been performed in accordance with the Declaration of Helsinki and approved by the Research Ethics Committee of Taichung Tzu Chi Hospital (REC111-04), which waived the need for informed consent due to the retrospective nature of the study.

### Design of the study and patient population

This retrospective cohort study was conducted at a regional teaching hospital in Taiwan from January 2009 to October 2021. The inclusion criteria were as follows: (1) patients with NSCLC with International Classification of Diseases, 10^th^ Revision, Clinical Modification (ICD-10-CM) codes C34.0-C34.9; (2) patients who were prescribed EGFR-TKIs (gefitinib or erlotinib) and treated for at least 1 week; (3) patients with cytologically or histologically confirmed stage IIIA, IIIB, or IV NSCLC who presented with EGFR mutations based on the Study of Lung Cancer (8^th^ edition) of the Tumor Node Metastasis Staging classification; and (4) patients aged > 20 years. Patients whose electronic medical records were incomplete were excluded. The index date in the study group was defined as the day when EGFR-TKI was first prescribed.

### TC and PPI exposure

The TC and PPI period was calculated from the index date until the conclusion of the study.

Patients were divided into the following four groups: TC-/PPI-, TC + /PPI-, TC-/PPI + , and TC + /PPI + .

- (1) The TC-/PPI- group consisted of patients who were neither on TC nor PPI therapy (control group).
- (2) The TC + /PPI- group consisted of patients whose period of TC (doxycycline or minocycline) and EGFR-TKI therapy overlapped by at least 20% but were not on PPIs.
- (3) The TC-/PPI + group consisted of patients whose period of PPI (esomeprazole, pantoprazole, rabeprazole, or lansoprazole) and EGFR-TKI therapy overlapped by at least 20% but were not on TCs.
- (4) The TC + /PPI + group consisted of patients taking TCs and PPIs concomitantly with EGFR-TKI therapy, and both therapeutic regimens overlapped by at least 20%.

### Statistical analysis

PFS was the primary endpoint of this study, while OS was the secondary endpoint. PFS was defined by the time interval between EGFR-TKI initiation to disease progression or death, whereas OS was defined by the time interval between EGFR-TKI initiation and death. The day of EGFR-TKI initiation was designated as the index date. Patients were followed-up until an endpoint was reached or until January 10, 2022.

The chi-squared test or Fisher's exact test was used to compare the categorical variables, and the Kruskal–Wallis test or analysis of variance was used for the continuous variables. The Cox proportional-hazards model was employed to analyze all selected variables related to the clinical outcomes. All statistical analyses were conducted using SAS v9.3 (SAS Institute Inc., Cary, NC, USA) and SPSS v28 (IBM Corp., Armonk, NY, USA) statistical software. P-values < 0.05 were considered statistically significant for all analyses. In this study, the Cox regression model was devised with a 0.95 overall probability of death based on a sample of 249 (TC-/PPI- plus TC-/PPI +) observations, which achieved more than 90% power to detect a regression coefficient of 1.70 at a 0.05 significance level.

## Results

**Fig. 1:**
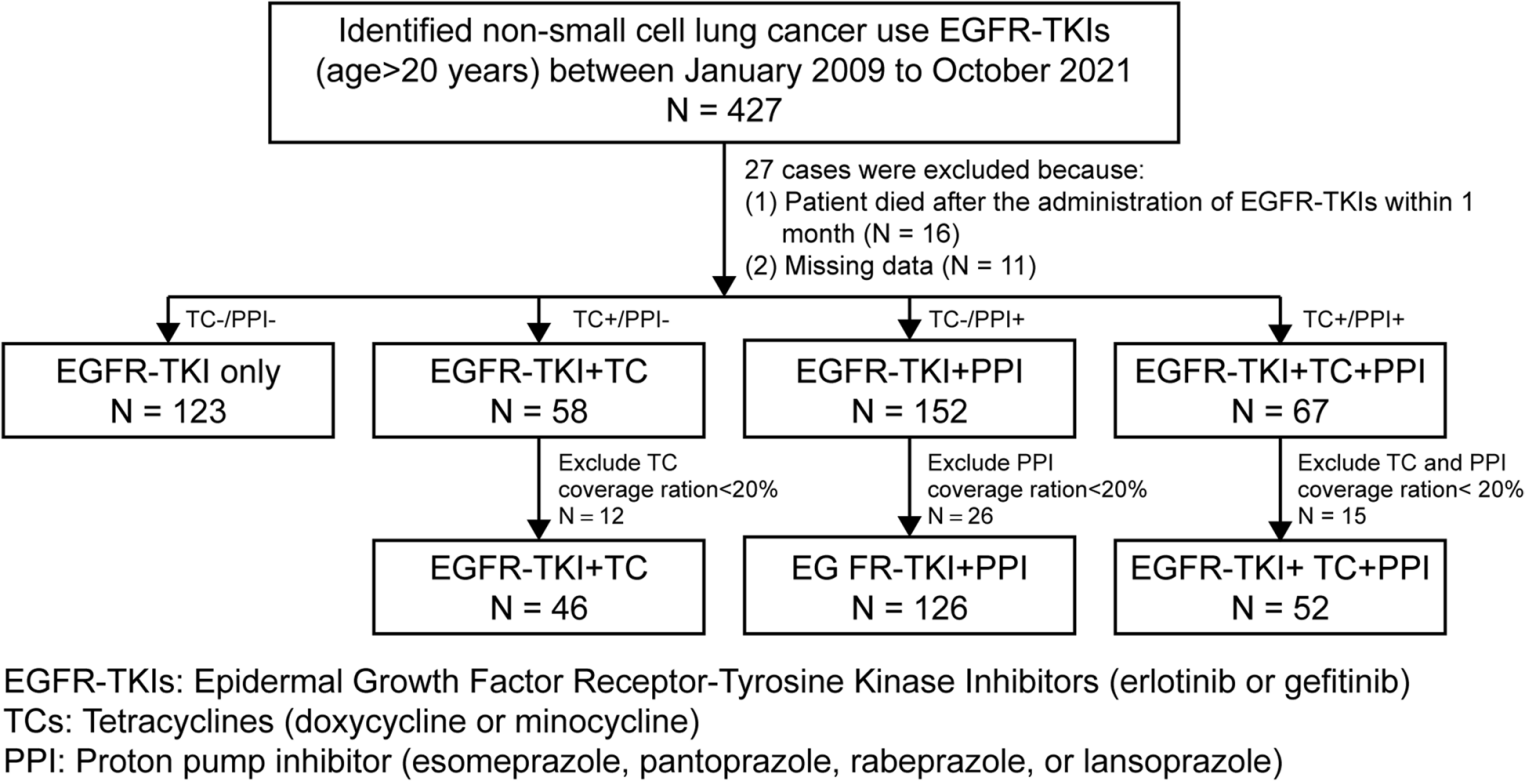
Study flow diagram EGFR-TKIs, epidermal growth factor receptor tyrosine kinase inhibitors

A total of 427 patients were diagnosed with NSCLC based on the ICD-10-CM system and categorized as C34.0–C34.9 between January 2009 and October 2021. Ultimately, 347 patients (136 men and 211 women) who met the study’s eligibility criteria were enrolled. The participants’ mean age was 65.3 ± 11.9 years (Fig. 1). The TC-/PPI-, TC + /PPI-, TC-/PPI + , and TC + /PPI + groups comprised 123 (35.4%), 46 (13.3%), 126 (36.3%), and 52 (15.0%) patients, respectively. Most patients (*n* = 314, 90.49%) had stage IV cancer at baseline, and the rest had stage IIIA or IIIB NSCLC. Brain metastases were not observed in 61.67% (*n* = 214) of patients, and 51.87% (*n* = 180) had an Eastern Cooperative Oncology Group (ECOG) Performance Status score of 0–1 at the beginning of treatment with TKIs. Adenocarcinoma was the most common histopathologic subtype of NSCLC (*n* = 332, 95.68%), followed by squamous cell carcinoma (*n* = 15, 4.32%). Exon 19 deletions were detected in 142 (40.92%) patients, while 205 (59.08%) patients had exon 21 (L858R) mutations. Erlotinib was used to treat 137 (84.05%) patients, while 26 (15.95%) patients were treated with gefitinib. Progressive disease (PD) was observed in 46.11% (*n* = 160) of patients. Skin rash was observed in 66.28% (*n* = 230) of patients. TC prophylactic use (before initiation of rash) was observed in 7.14% (*n* = 7) of patients and later use (rash management) was observed in 92.86% (*n* = 91) of patients. ECOG Performance Status score, brain metastases, PD, and skin rash were the only baseline characteristics exhibiting statistically significant differences among the four groups (Table 1).

**Table 1:** Characteristics of the study population

	**Group**								
	**TC-/PPI- *n* = 123**		**TC + /PPI- *n* = 46**		**TC-/PPI + *n* = 126**		**TC + /PPI + *n* = 52**		
Variable	**n**	**%**	**n**	**%**	**n**	**%**	**n**	**%**	***p*-value**
**Age, years (mean ± SD)**	65.6 ± 11.3	-	63.2 ± 11.0	-	65.6 ± 12.8	-	66.0 ± 12.2	-	0.399
**Sex (male)**	46	37.4	18	39.1	43	34.1	29	55.8	0.057
**Smoking**	38	30.9	14	30.4	28	22.2	19	36.5	0.211
**Family history of cancer**	11	8.9	10	21.7	10	7.9	5	9.6	0.058
**ECOG Performance Status score**									0.006^*^
0	30	24.4	19	41.3	20	15.9	13	25.0	
1	70	56.9	24	52.2	65	51.6	29	55.8	
2	21	17.1	3	6.5	29	23.0	9	17.3	
3	2	1.6	0	0.0	11	8.7	1	1.9	
4	0	0.0	0	0.0	1	0.8	0	0.0	
**Stage of cancer**									0.376
IIIA	1	0.8	0	0.0	2	1.6	2	3.8	
IIIB	12	9.8	6	13.0	7	5.6	3	5.8	
IV	110	89.4	40	87.0	117	92.9	47	90.4	
**Histopathology**									0.824
Adenocarcinoma	117	95.1	45	97.8	121	96.0	49	94.2	
Squamous cell carcinoma	6	4.9	1	2.2	5	4.0	3	5.8	
**Number of metastases**									0.590
0	13	10.6	6	13.0	9	7.1	5	9.6	
1	56	45.5	20	43.5	50	39.7	21	40.4	
2	40	32.5	12	26.1	38	30.2	17	32.7	
≥ 3	14	11.4	8	17.4	29	23.0	9	17.3	
**Brain metastases**	40	32.5	12	26.1	60	47.6	21	40.4	0.025^*^
**EGFR mutation status**									0.769
Exon 19 deletion	48	39.0	17	37.0	56	44.4	21	40.4	
Exon 21 L858R	75	61.0	29	63.0	70	55.6	31	59.6	
**EGFR-TKI**									0.919
Erlotinib	65	52.8	26	56.5	67	53.2	30	57.7	
Gefitinib	58	47.2	20	43.5	59	46.8	22	42.3	
**Progressive disease**	64	52.0	27	58.7	39	31.0	30	57.7	< 0.001*
**Skin rash**	73	59.3	46	100.0	59	46.8	52	100.0	< 0.001*
**TC group**									
Prophylaxis use			4	8.7			3	5.8	
Later use			42	91.3			49	94.2	

*Abbreviations:* *SD* standard deviation, *TC* tetracyclines, *PPI* proton pump inhibitor, *ECOG* Eastern Cooperative Oncology Group, *EGFR-TKI* epidermal growth factor receptor tyrosine kinase inhibitor

^*^ *P-value* < 0.05

### Primary endpoints

**Fig. 2:**
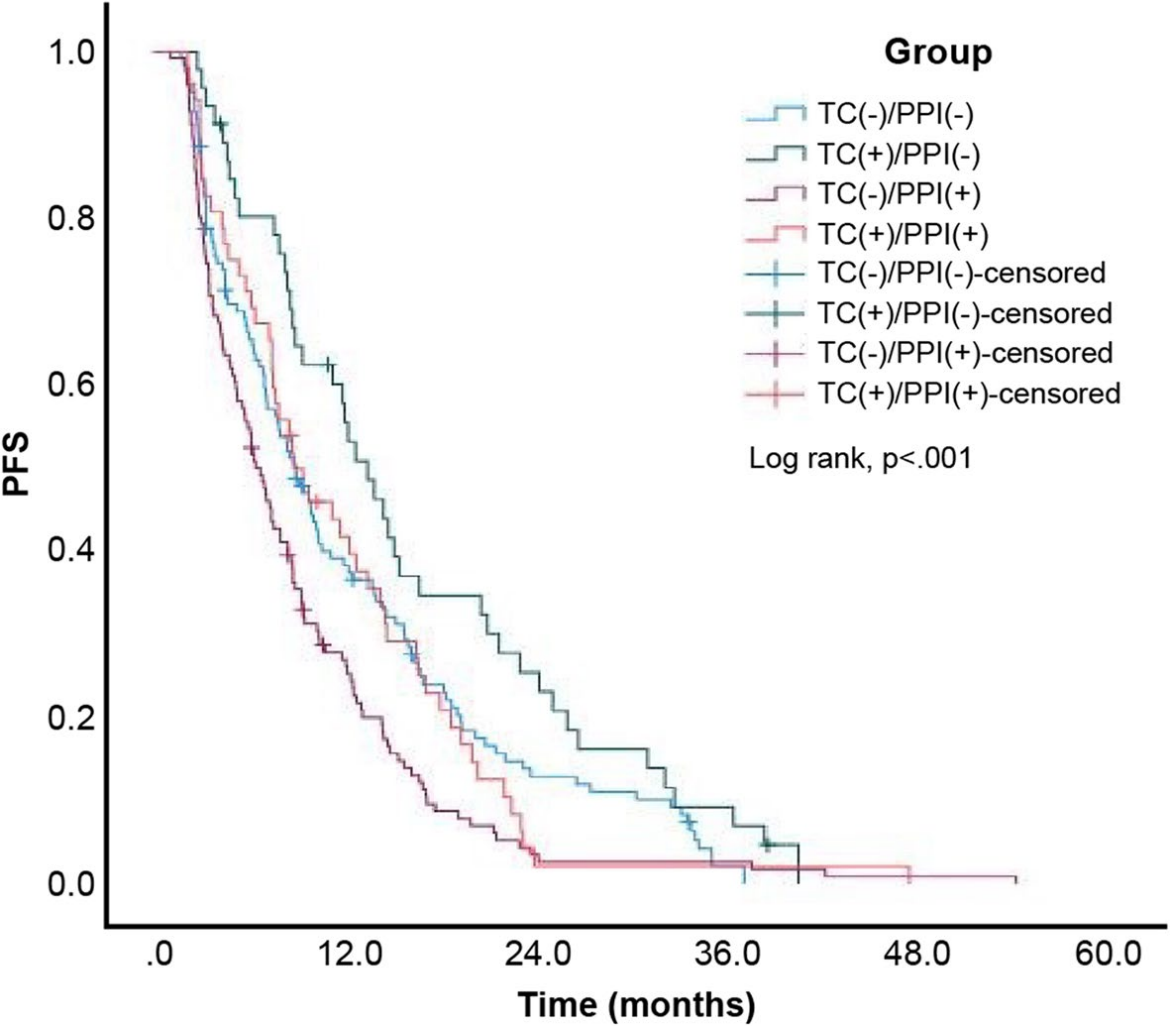
Kaplan–Meier curves of PFS according to TC and/or PPI use. PFS: progression-free survival, TC: tetracycline, PPI: proton pump inhibitor

**Fig. 3:**
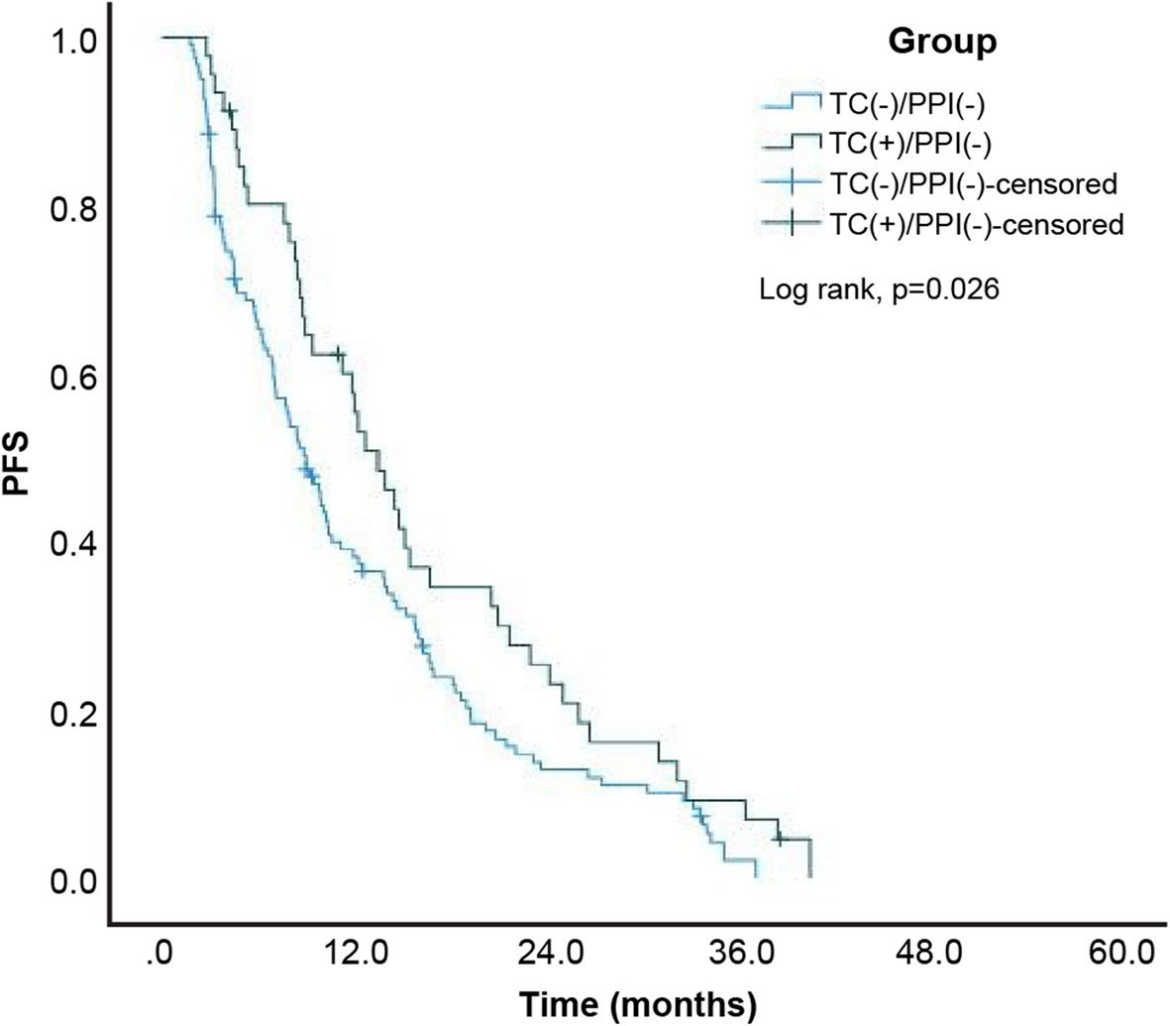
Kaplan–Meier curves of PFS for TC + /PPI- and TC-/PPI-. PFS: progression-free survival, TC: tetracycline, PPI: proton pump inhibitor

**Fig. 4:**
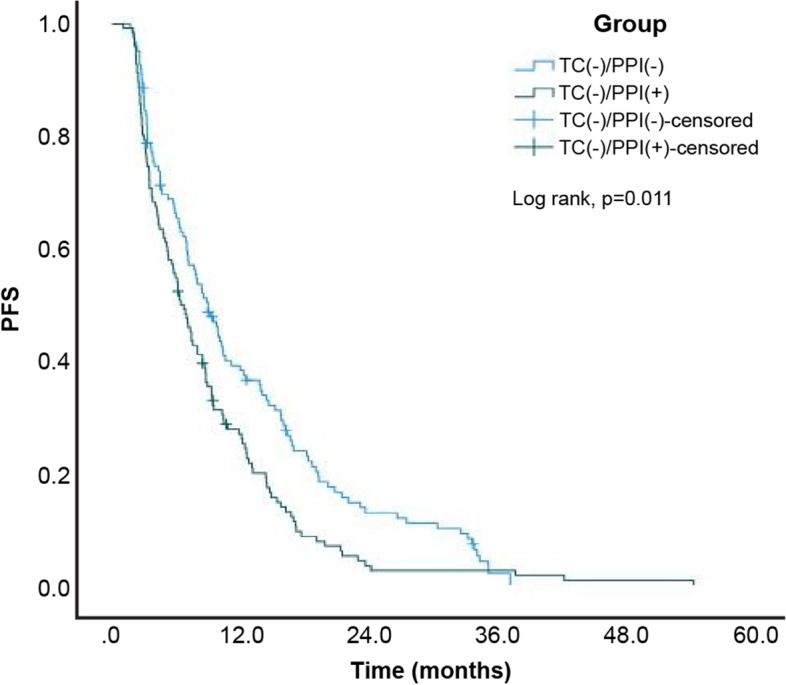
Kaplan–Meier curves of PFS for TC-/PPI + and TC-/PPI-. PFS: progression-free survival, TC: tetracycline, PPI: proton pump inhibitor

**Fig. 5:**
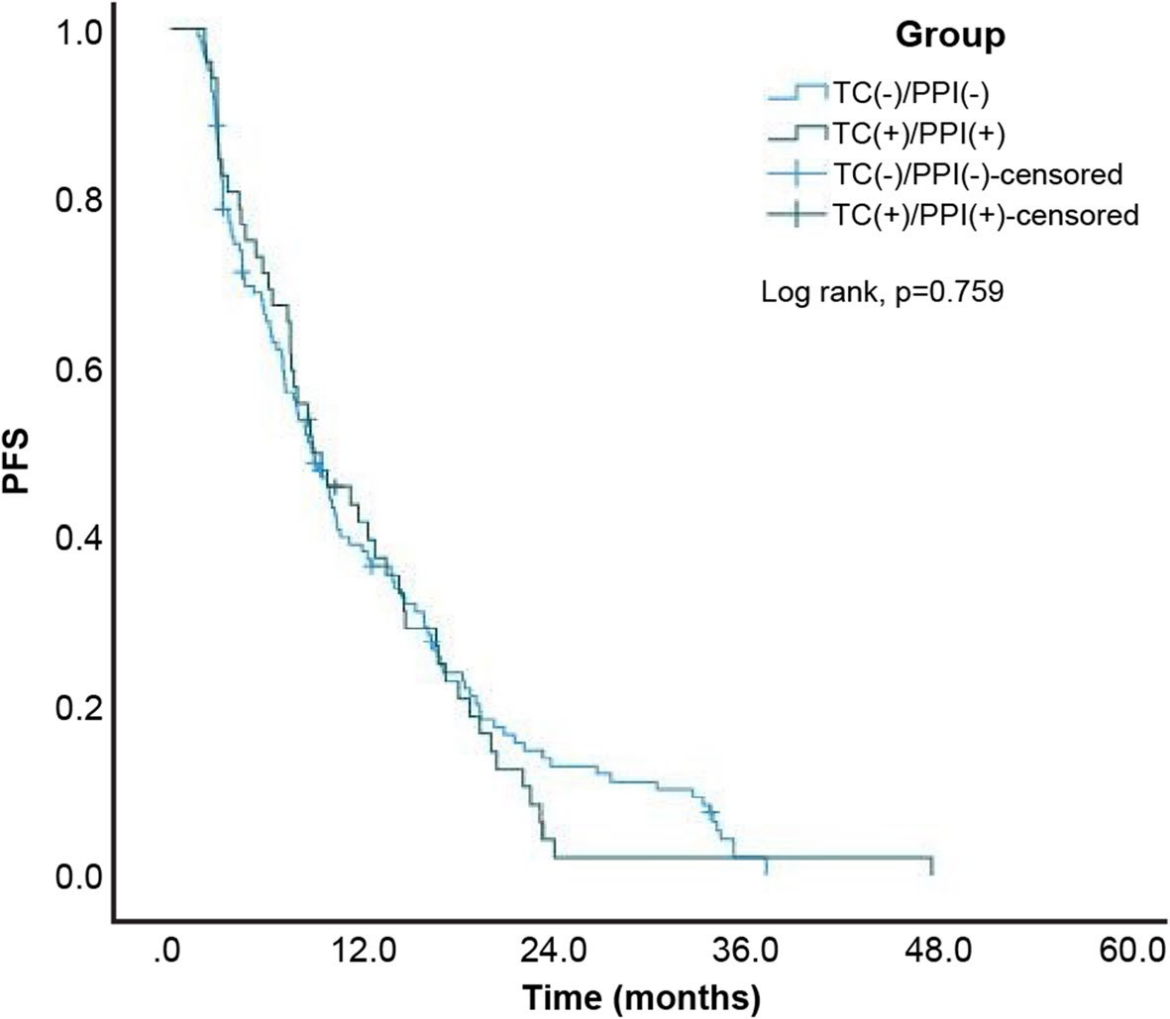
Kaplan–Meier curves of PFS for TC + /PPI + and TC-/PPI-. PFS: progression-free survival, TC: tetracycline, PPI: proton pump inhibitor

A total of 330 (95.1%) patients among the 347 patients exhibited disease progression or died at the end of the study. The estimated median PFS of the entire study cohort was 8.57 (95% confidence interval [CI]: 7.66–9.48) months. Moreover, the estimated median PFS for patients in the TC-/PPI-, TC + /PPI-, TC-/PPI + , and TC + /PPI + groups was 9.00 (95% CI: 7.28–10.72), 13.53 (95% CI: 10.39–16.67), 6.43 (95% CI: 5.23–7.64), and 8.83 (95% CI: 5.00–12.66) months, respectively. The Kaplan–Meier cumulative rate for PFS differed significantly (log rank test: *p* < 0.001) among the four groups (Fig. 2). Subgroup analysis revealed statistically significant differences between the TC + /PPI- and TC-/PPI- groups (*p* = 0.026; Fig. 3), and between the TC-/PPI + and TC-/PPI- groups (*p* = 0.011; Fig. 4). The PFS of the TC + /PPI + group did not differ significantly from that of the TC-/PPI- group (*p* = 0.759; Fig. 5). We used Cox proportional-hazards models to analyze the multivariate data, which yielded the HR of PFS associated with the use of TC and/or PPI and other related variables such as age, sex, ECOG Performance Status score, brain metastases, and skin rash. The results were statistically significant for ECOG Performance Status score = 1, ECOG Performance Status score ≥ 2, brain metastases, and skin rash. The HRs for the association of PFS with ECOG Performance Status score = 1, ECOG Performance Status score ≥ 2, brain metastases and skin rash were 1.58 (95% CI: 1.18–2.11; *p* = 0.002), 2.36 (95% CI: 1.65–3.36; *p* < 0.001), 1.60 (95% CI: 1.25–2.04; *p* < 0.001) and 0.35 (95% CI: 0.26–0.47; *p* < 0.001), respectively. After adjusting for age, sex, ECOG Performance Status score, brain metastases and skin rash, the HR was higher in the TC + /PPI- (HR 1.10, 95% CI: 0.75–1.61; *p* = 0.615), TC-/PPI + groups (HR 1.29, 95% CI: 0.99–1.68; *p* = 0.062), and TC + /PPI + group (HR: 1.37, 95% CI: 0.95–1.98; *p* = 0.097) compared to the TC-/PPI- group, and none of these differences retained statistical significance (Table 2).

**Table 2:** Cox proportional-hazards model evaluating the effect of the clinical variables on progression-free survival

	Crude			Adjusted^†^		
Variable	HR	(95% CI)	*p-*value	HR	(95% CI)	*p-*value
**Age (every one year)**	1.00	0.99–1.01	0.552	1.00	0.99–1.01	0.904
**Sex (male vs. female)**	1.42	1.13–1.77	0.003^*^	1.21	0.96–1.54	0.108
**ECOG PS**						
0	Ref			Ref		
1	1.82	1.39–2.39	< 0.001^*^	1.58	1.18–2.11	0.002^*^
≥ 2	2.76	1.99–3.82	< 0.001^*^	2.36	1.65–3.36	< 0.001^*^
**Group**						
TC-/PPI-	Ref			Ref		
TC + /PPI-	0.70	0.49–0.99	0.044^*^	1.10	0.75–1.61	0.615
TC-/PPI +	1.43	1.11–1.86	0.006^*^	1.29	0.99–1.68	0.062
TC + /PPI +	1.05	0.75–1.48	0.776	1.37	0.95–1.98	0.097
**Brain metastases**	2.01	1.59–2.56	< 0.001^*^	1.60	1.25–2.04	< 0.001^*^
**Skin rash**	0.32	0.25–0.41	< 0.001^*^	0.35	0.26–0.47	< 0.001^*^

*Abbreviations:* *HR* hazard ratio, *ECOG* Eastern Cooperative Oncology Group, *PS* performance status, *TC* tetracycline, *PPI* proton pump inhibitor

^†^Adjusted for age, sex, ECOG PS, and brain metastases

^*^ *P-value* < 0.05

### Secondary endpoints

**Fig. 6:**
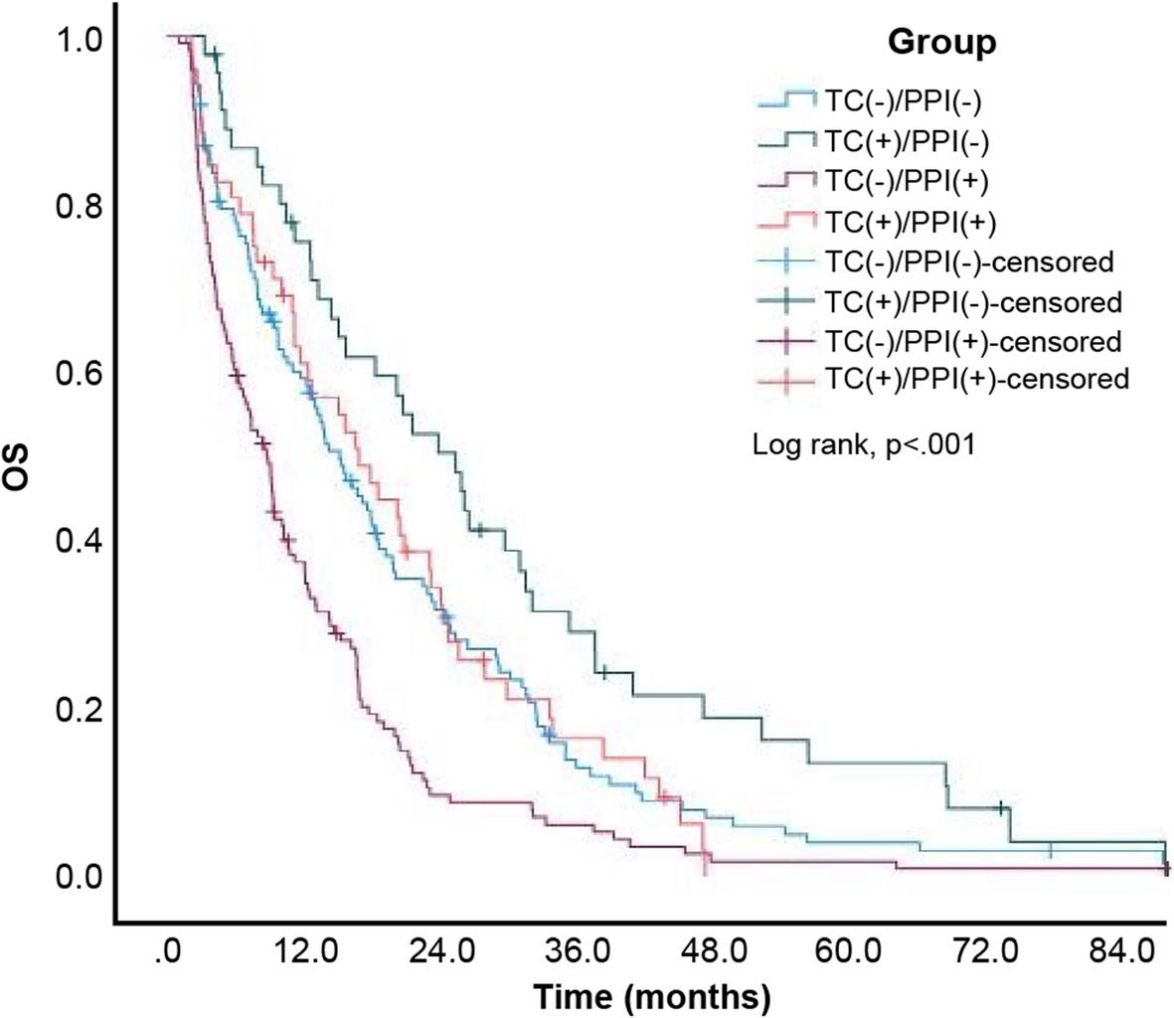
Kaplan Meier curves of OS according to TC and/or PPI use. OS: overall survival, TC: tetracycline, PPI: proton pump inhibitor

**Fig. 7:**
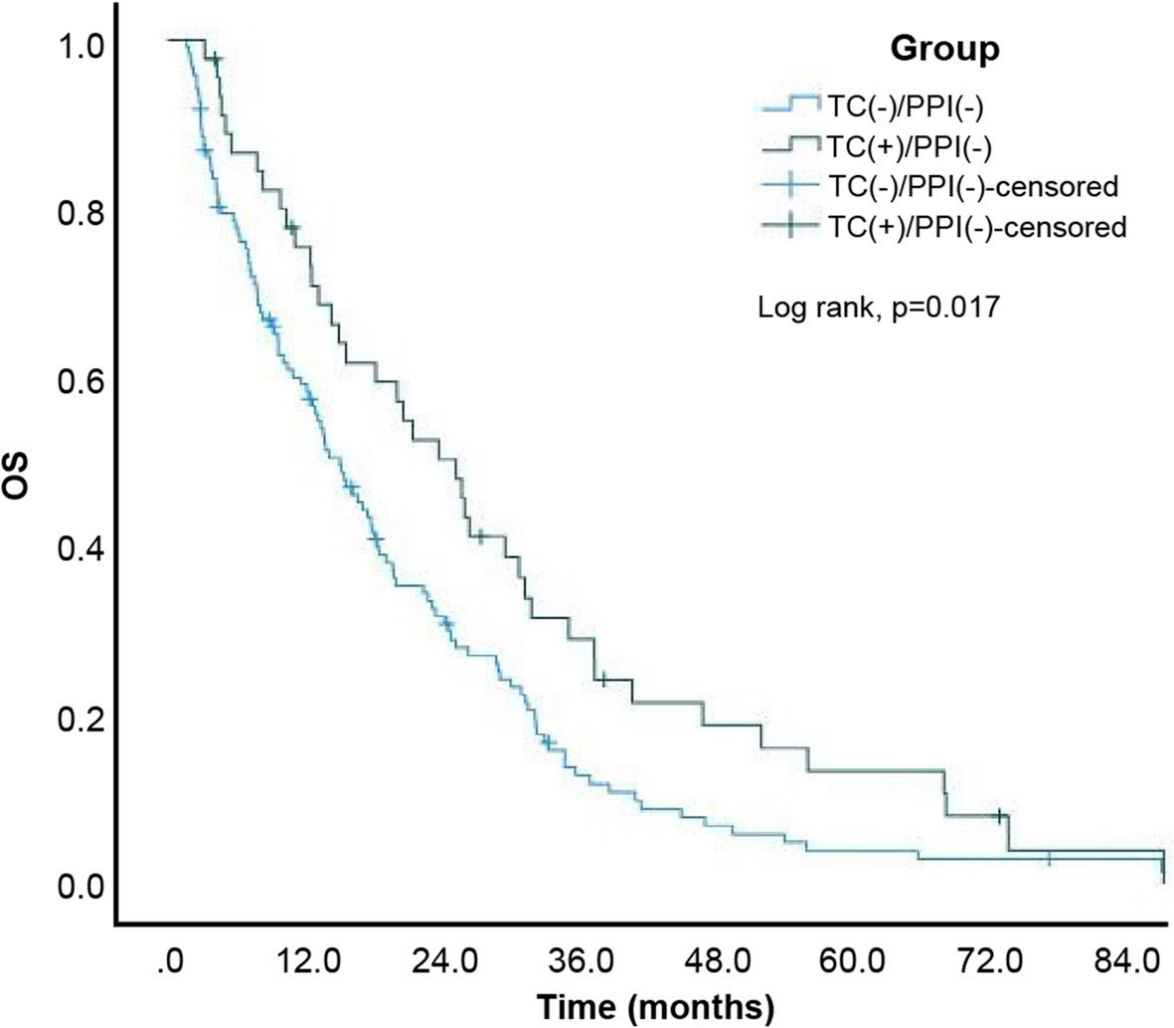
Kaplan Meier curves of OS for TC + /PPI- and TC-/PPI-. OS: overall survival, TC: tetracycline, PPI: proton pump inhibitor

**Fig. 8:**
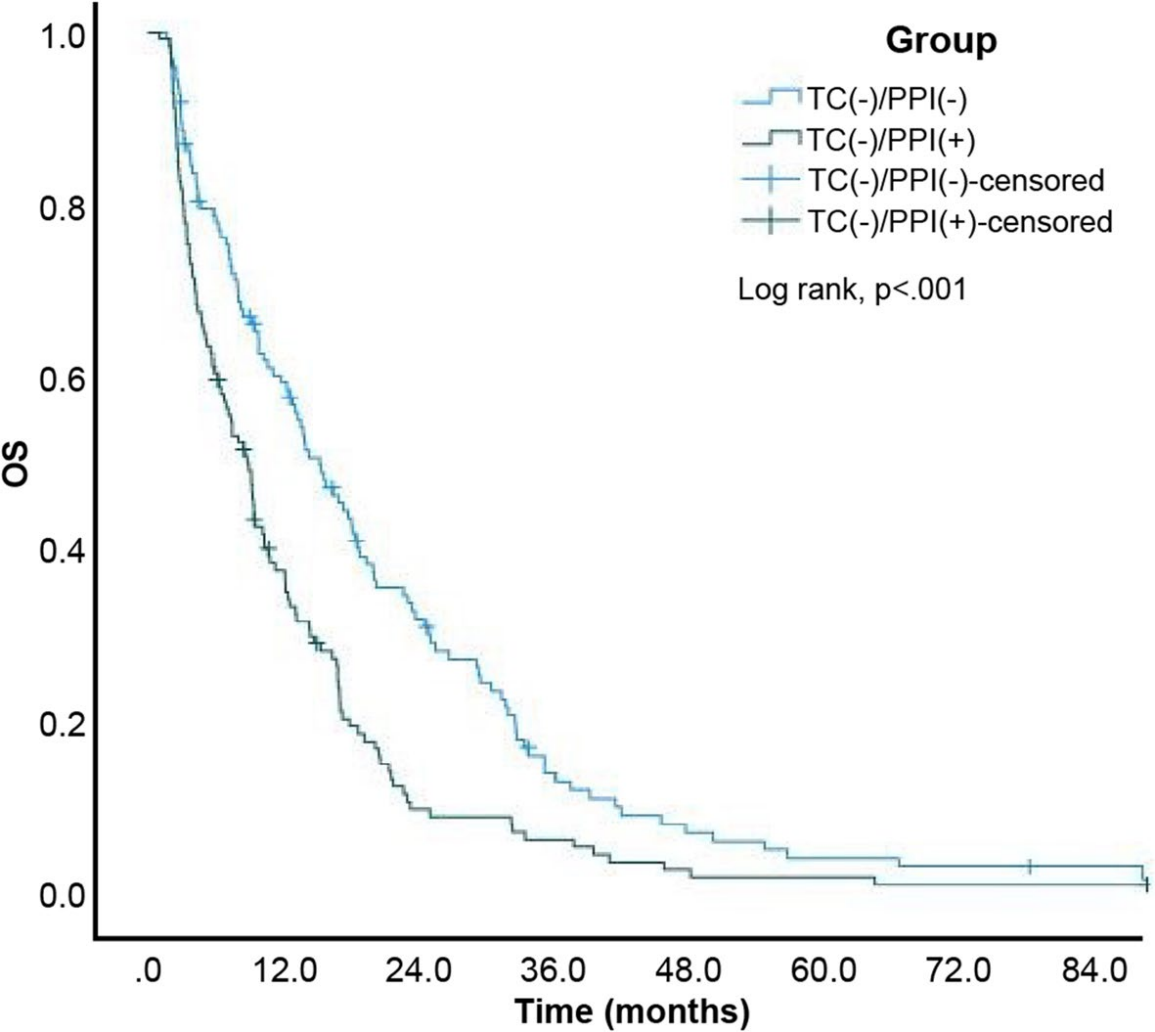
Kaplan Meier curves of OS for TC-/PPI + and TC-/PPI-. OS: overall survival, TC: tetracycline, PPI: proton pump inhibitor

**Fig. 9:**
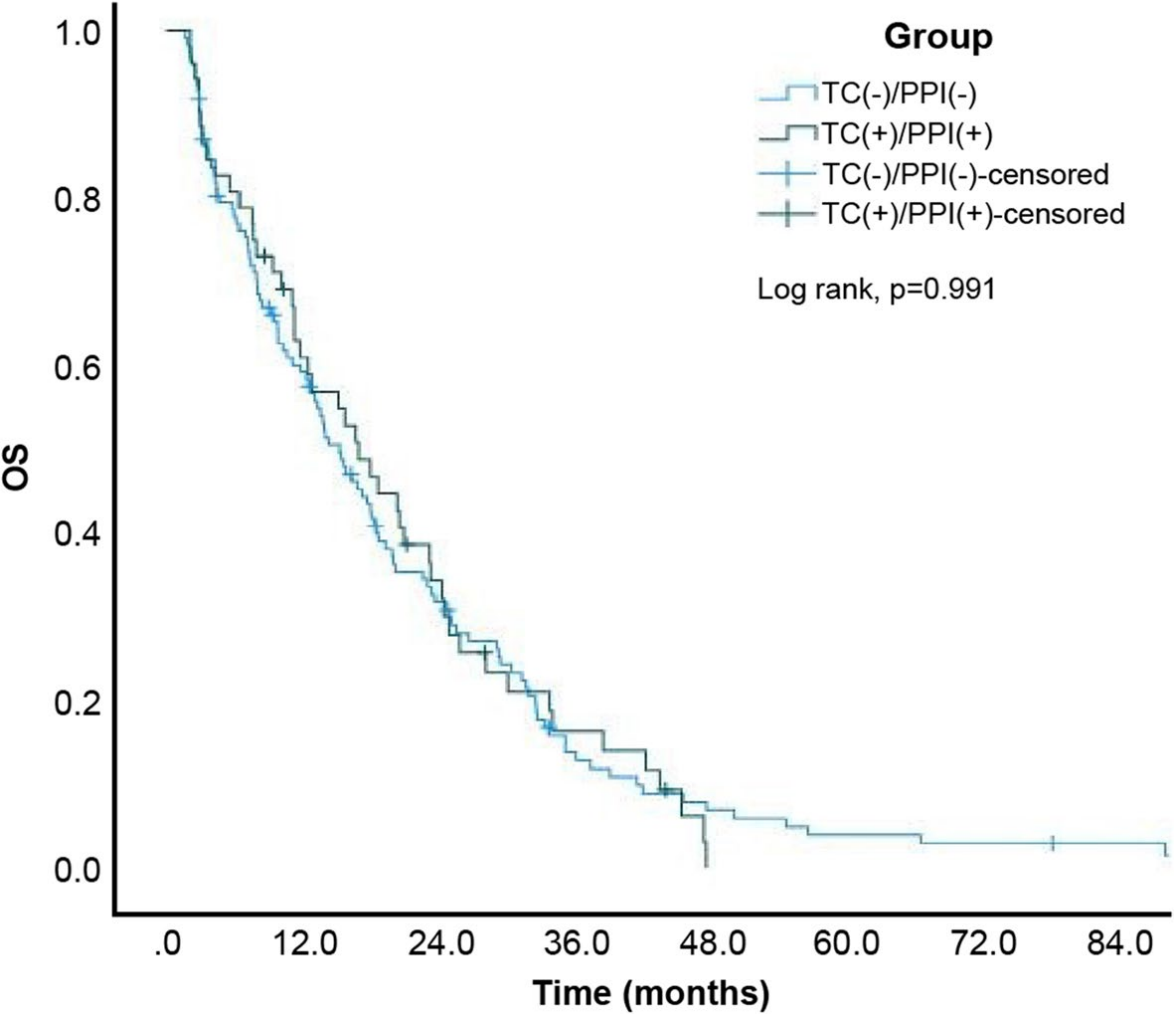
Kaplan Meier curves of OS for TC + /PPI + and TC-/PPI-. OS: overall survival, TC: tetracycline, PPI: proton pump inhibitor

Eventually, 320 (92.2%) of 347 patients died. The estimated median OS of the entire study cohort was 13.10 (95% CI: 11.03–15.17) months. Moreover, the estimated median OS of patients from the TC-/PPI-, TC + /PPI-, TC-/PPI + , and TC + /PPI + groups was 15.30 (95% CI: 11.97–18.63), 25.60 (95% CI: 19.15–32.05), 8.80 (95% CI: 6.95–10.65), and 17.00 (95% CI: 10.28–23.72) months, respectively. The Kaplan–Meier cumulative rate for OS differed significantly (log rank, p < 0.001) among the 4 groups (Fig. 6). Subgroup analysis revealed statistically significant differences between the TC + /PPI- and TC-/PPI- groups (*p* = 0.017; Fig. 7) and between the TC-/PPI + and TC-/PPI- groups (*p* < 0.001; Fig. 8), respectively. The OS of the TC + /PPI + group did not differ significantly from that of the TC-/PPI- group (*p* = 0.991; Fig. 9). The results of the multivariable hazards model were statistically significant for sex, ECOG PS = 1, ECOG PS ≥ 2, brain metastases and skin rash with HR values for OS equaling 1.29 (95% CI: 1.02–1.64; *p* = 0.034), 1.80 (95% CI: 1.33–2.42; *p* < 0.001), 2.82 (95% CI: 1.94–4.10; *p* < 0.001), 2.11 (95% CI: 1.62–2.73; *p* < 0.001) and 0.28 (95% CI: 0.21–0.37; *p* < 0.001), respectively. After adjusting for covariables with the TC-/PPI- group as a reference, the adjusted HR of the TC-/PPI + group was 1.50 (95% CI: 1.15–1.96; *p* = 0.003) (Table 3).

**Table 3:** Cox proportional hazards model evaluating the effect of clinical variables on overall survival

	Crude			Adjusted^†^		
Variable	HR	(95% CI)	*p*-value	HR	(95% CI)	*p-*value
**Age (every one year)**	1.01	1.00–1.02	0.028^*^	1.01	0.99–1.02	0.121
**Sex (male vs. female)**	1.31	1.05–1.64	0.019^*^	1.29	1.02–1.64	0.034^*^
**ECOG PS**						
0	Ref			Ref		
1	2.08	1.57–2.75	< 0.001^*^	1.80	1.33–2.42	< 0.001^*^
≥ 2	3.79	2.69–5.34	< 0.001^*^	2.82	1.94–4.10	< 0.001^*^
**Group**						
TC-/PPI-	Ref			Ref		
TC + /PPI-	0.65	0.45–0.93	0.019^*^	1.07	0.73–1.59	0.708
TC-/PPI +	1.70	1.31–2.20	< 0.001^*^	1.50	1.15–1.96	0.003^*^
TC + /PPI +	0.99	0.70–1.39	0.931	1.30	0.89–1.88	0.175
**Brain metastases**	2.24	1.75–2.86	< 0.001^*^	2.11	1.62–2.73	< 0.001^*^
**Skin rash**	0.25	0.19–0.32	< 0.001*	0.28	0.21–0.37	< 0.001^*^

*Abbreviations:* *HR* hazard ratio, *CI* confidence interval, *ECOG* Eastern Cooperative Oncology Group, *TC* tetracycline, *PPI*, proton pump inhibitor

^†^Adjusted for age, sex, ECOG Performance Status, and brain metastases

^*^ *P-value* < 0.05

## Discussion

PPIs increase the gastric pH, which may decrease TKI’s solubility, leading to decreased absorption, although the evidence of this clinical effect is limited. A retrospective study conducted in 2018 analyzed the impact of gastric acid inhibitors on the clinical outcomes of patients with NSCLC treated with EGFR-TKIs. The study showed that the mean PFS was 84 days (95% CI, 65–101) and 221 days (95% CI, 125–429; *p* < 0.0001) with or without the use of gastric acid suppressants, respectively. Concomitant use of gastric acid inhibitors and TKIs may cause adverse PFS outcomes in patients with NSCLC, irrespective of the type of gastric acid inhibitor used [16]. A retrospective study conducted in 2021 evaluated the results of a randomized phase III (ARCHER 1050) study on the effect of concurrent administration of PPIs on the PFS and OS in patients treated with dacomitinib or gefitinib. The results showed that the co-administration of PPIs with dacomitinib or gefitinib had no effect on the PFS or OS. PPI use was associated with reduced absorption of gefitinib or dacomitinib in pharmacokinetic studies; however, the use of PPIs in patients with NSCLC may have little effect on the efficacy of these anti-cancer drugs [17]. Evidence on concurrent usage of EGFR-TKIs and PPIs and their effect on the efficacy of the former is inconsistent. Our study’s median PFS and OS were 9.00 and 15.30 months in the TC-/PPI- group, respectively, and 6.43 and 8.8 months in the TC-/PPI + group, respectively, i.e., differences between the two groups were statistically significant regardless of the erlotinib or gefitinib treatment. Concomitant use of EGFR-TKIs and PPIs may adversely affect the PFS and OS outcomes. Concomitant use of PPIs can directly reduce the effectiveness of erlotinib/gefitinib due to a decrease in their absorption.

Skin rashes caused by EGFR-TKIs can be relieved by TCs. According to a prospective, randomized phase III study (*N* = 150) conducted in 2016, the incidence of skin toxicity caused by erlotinib was 84%. The OS was longer for patients whose rashes were treated with minocycline (8 months) than for those who were not treated for rash (6 months) [18]. A retrospective study conducted in 2020 found that TCs were associated with better survival rates for patients treated with erlotinib; this demonstrated the benefit of administering TCs with erlotinib [HR: 0.68 (95% CI: 0.58–0.78)]. However, the gefitinib users did not derive additional benefits from the use of TCs, suggesting that the benefit of an improved OS due to TCs may be owing to the relief of rash and continued treatment with TKIs [19]. In our study, the results were similar to the findings proposed by Melosky et al. [18]. Skin rash developed in 66.28% (*n* = 230) of patients. TC prophylactic and its later use was observed in 7.14% (*n* = 7) and 92.86% (*n* = 91) of patients respectively. The estimated median PFS and OS were 9.00 and 15.30 months in the TC-/PPI- group, respectively, and 13.53 and 25.6 months in the TC + /PPI- group, respectively, with significant differences between these two groups. Thus, concomitant use of EGFR-TKIs and TCs can improve PFS and OS of NSCLC patients. It is noteworthy that patients who develop skin rash due to EGFR-TKIs are more likely to respond to the treatment, and rash has been found to be an independent predictive factor for improved survival. Skin toxicity could be a potential indicator of efficacy. Therefore, we suggest that TCs used for relieving skin rash indirectly affect the patients’ survival because they are used mainly in a subgroup of patients with skin toxicity and better prognosis. The TCs benefit may be due to the development of patients’ skin toxicity and not due to the TC itself.

To date, no study has analyzed and explored the effect of the concomitant use of TCs and PPIs on the PFS or OS in patients receiving EGFR-TKI therapy. A 2011 review article linked the drugs containing metal cations (e.g. antacids Al3 + and Mg2 +) produced by chemical chelating interactions with oral TCs (e.g. tetracycline, doxycycline). As reported, the concomitant administration of antibacterial tetracycline and antacid aluminum hydroxide or magnesium hydroxide can cause the chelating drug-drug interaction (DDI) to reduce tetracycline oral bioavailability by 80%. It is noteworthy that this interaction was not observed with the concomitant administration of PPI or histamine H2-receptor antagonists with oral TCs in this review article; however, the mechanism of DDI was explained based on chelation rather than elevated gastric pH [20]. In another study, the pKa' = 3.09 for protonation of doxycycline was determined spectrophotometrically [21]. A clinical trial in 1994 reported that doxycycline is absorbed in the duodenum, where the pH is approximately 5–7, in its unionized form. Therefore, the use of PPIs may cause impaired absorption of TCs [22]. Evidence on DDI and concomitant use of PPIs and TCs is inconsistent. Our study found that the estimated median PFS and OS were 9.00 and 15.30 months for the TC-/PPI- group, respectively, and 8.83 and 17.00 months for the TC + /PPI + group, respectively, without any significant differences between the two groups. Concomitant use of EGFR-TKIs, TCs, and PPIs did not decrease the PFS and OS compared to EGFR-TKI therapy alone.

Several limitations of this retrospective cohort study must be considered. The first and principal limitation was that the clinical data and medical records were extracted from the hospital information system; thus, physician-related personal factors may be responsible for incomplete records such as the severity of skin toxicity. Second, the sample size was small, especially for the TC + /PPI- and TC + /PPI + subgroups, which did not permit an accurate assessment of the treatment response.

## Conclusions

The concomitant use of EGFR-TKIs and PPIs decreased the PFS and OS in patients with NSCLC. Concomitant use of PPIs can directly reduce the effectiveness of erlotinib/gefitinib due to a decrease in their absorption. Conversely, concomitant use of EGFR-TKIs and TCs improved PFS and OS in patients with NSCLC. The TCs benefit may be due to the development of patients’ skin toxicity and not due to the TC itself However, the effect of the concomitant use of EGFR-TKIs, PPIs, and TCs on the PFS and OS was similar to that of EGFR-TKI therapy alone. We conclude that the possible interactions between EGFR-TKIs and other drugs pose a significant challenge to managing patients treated with the former. Therefore, it is important to reassess the indication and necessity of PPI or TC therapy in patients receiving erlotinib or gefitinib by weighing the benefits and risks of possible discontinuation due to the clinical relevance of this interaction.

## Data Availability

All data generated or analyzed during this study are included in this published article.

## Abbreviations

CI: Confidence interval
ECOG: Eastern Cooperative Oncology Group
EGFR-TKIs: Epidermal growth factor receptor tyrosine kinase inhibitors
HR: Hazard ratio
NSCLC: Non-small cell lung cancer
OS: Overall survival
PFS: Progression-free survival
PPI: Proton pump inhibitor
TC: Tetracycline
DDI: Drug-drug interaction
